# Occurrence, Risk Factors, and Antibiotic Resistance Patterns of *Salmonella* Isolates Obtained From Dairy Farms in Gondar Town, Northwest Ethiopia

**DOI:** 10.1155/bmri/7778947

**Published:** 2025-01-28

**Authors:** Tsedalu Yirsa, Alene Tigistu

**Affiliations:** ^1^Department of Veterinary Medicine, College of Agriculture, Woldia University, Woldia, Ethiopia; ^2^Department of Veterinary Medicine, College of Veterinary Medicine and Animal Sciences, Samara University, Samara, Ethiopia

**Keywords:** antibiotic susceptibility, dairy farm, Gondar town, *Salmonella*

## Abstract

**Background:** Salmonellosis is a foodborne diarrheal disease with significant public health and economic implications for humans and animals. Nevertheless, its epidemiological effects and multidrug resistance remain poorly studied in the study area.

**Methods:** A cross-sectional study was conducted from March to August 2022 to determine the occurrence, risk factors, and antibiotic susceptibility patterns of *Salmonella* isolates in the study dairy farms. A total of 232 samples (200 study animals and 32 bulk tank milk) were collected from purposively selected dairy farms in the study area. Again, fecal and bulk tank milk samples were collected, pre-enriched, and cultured to examine the presence of *Salmonella* following standard techniques. The pure 30 isolates were also subjected to a Kirby–Bauer disk diffusion test on Muller–Hinton agar to assess their antibiotic susceptibility patterns. Binary logistic regression analysis in Statistical Package for Social Sciences (SPSS) Version 25 was used to determine the strength of the risk factors associated with the occurrence of this disease.

**Findings:** The overall prevalence of *Salmonella* isolates was 12.9% (30/232) across all samples. Among these, 11.5% (23/200) of the fecal samples from the study animals and 21.9% (7/32) of the bulk tank milk samples tested positive for this disease. A statistically significant association was observed between the presence of the *Salmonella* isolates and the age of the study animals, farm size, bedding, and udder washing practices. The isolates also showed the highest resistance to ampicillin (100%) and tetracycline (96.7%). Conversely, the isolates were most sensitive to ciprofloxacin (100%) and kanamycin (90%).

**Conclusions:** Generally, the high presence of *Salmonella* isolates and its resistance to drugs pose significant economic and public health challenges. Thus, it is crucial to implement improved management practices and appropriate antibiotic therapies in the study dairy farms.

## 1. Introduction

Foodborne pathogens pose a considerable economic and public health challenge on a global scale [1]. Among these, salmonellosis stands out as a foodborne illness that predominantly impacts animals but can also be transmitted to humans [2]. This condition is caused by one or more of the 2500 serotypes of *Salmonella*, which are commonly present in animal-derived food products [3, 4]. Notably, *Salmonella* Enteritidis and *Salmonella* Typhimurium are often recognized as the leading foodborne *Salmonella* species across various countries [4]. *Salmonella* species comprises a group of gram-negative, rod-shaped, and facultative anaerobic bacilli that successfully infect various hosts [5, 6]. Cattle are identified as a major reservoir for *Salmonella* species, shedding the bacteria through their feces, which leads to the contamination of milk and other dairy products, thus creating a direct route for human infection with this disease [7].

Salmonellosis poses a significant threat, leading to considerable economic losses characterized by increased mortality rates, reduced livestock productivity, and potential transmission risks to humans [4, 8]. It is a major public health issue worldwide, with an estimated 93.8 million cases of gastroenteritis and around 155,000 deaths annually [9]. Furthermore, human salmonellosis is often associated with the consumption of contaminated animal products, direct contact with infected animals, and the use of contaminated equipment [6, 10]. The risk posed by asymptomatic carriers is particularly concerning, as livestock can transmit the pathogen to both the farm environment and surrounding areas, thereby heightening food safety risks [11]. The excretion of *Salmonella* in feces can result in the contamination of water, soil, other animals, and feed [6, 12]. Additionally, fecal matter from infected individuals and animals is a primary contributor to bacterial contamination in the environment and food supply, ultimately leading to infections [2, 13].

*Salmonella* species can enter a dairy farm through various routes, including human activity, contaminated feed or water, wild animals such as rodents and birds, and domesticated livestock [2]. Additionally, dairy cattle have been recognized as a possible source of antimicrobial-resistant (AMR) infections in humans, resulting from direct contact with the animals and the presence of AMR in raw milk, cheddar cheese, and hamburger meat derived from dairy farms [14]. The use of antimicrobials in livestock operations has been historically linked to the emergence and spread of AMR *Salmonella* [15]. Antimicrobial resistance poses an escalating threat to global public health, particularly in developing nations, where the increased availability of antibiotics without prescriptions and poor sanitation conditions contribute to the proliferation of resistant strains [16]. The occurrence of AMR *Salmonella* species is increasing, primarily due to administering antimicrobials in food-producing animals [4, 8, 9, 17, 18]. Using subtherapeutic levels or preventive doses not only promotes growth but also significantly raises the health risks associated with consuming contaminated dairy and meat products [19, 20]. The potential for milk and dairy products to act as a vehicle for antibiotic-resistant bacterial genes has generated considerable concern within the food industry, presenting a significant public health challenge [21].

Various studies have indicated the prevalence of isolated *Salmonella* and its antimicrobial susceptibility within veterinary and public health frameworks [2, 8, 12, 15, 19, 20, 22]. Nevertheless, a paucity of research focuses on seemingly healthy dairy cows and calves, as well as their handlers and equipment in the study area. Foodborne illnesses are also widespread in Ethiopia which is attributed to poor food handling and sanitation practices, inadequate food safety regulations, ineffective regulatory systems, and a lack of education among food handlers [1]. The screening of milk and other dairy products for pathogenic organisms is essential in mitigating human infections [4]. Investigating samples from dairy and calf farms is vital for developing strategies to minimize the potential transmission of *Salmonella* and the emergence of antibiotic-resistant strains between humans and livestock [8]. Consequently, this study seeks to identify *Salmonella* isolates, their associated risk factors, and antibiotic susceptibility in dairy cows and calves within the study area.

## 2. Methods and Materials

### 2.1. Study Area

This study was carried out from March to August 2022 in and around Gondar town, located in the Northwest Gondar zone of the Amhara National Regional State. Gondar is situated 748 km away from Addis Ababa, the capital city of the federal government of Ethiopia, and 120 km from Bahir Dar, the capital city of the Amhara National Regional State. The town covers a total area of 192.3 km^2^ and has a hilly terrain. As per the 2007 National Population and Housing Census, Gondar is positioned at an elevation ranging from 1500 to 2200 m above sea level, with a latitude and longitude of 12°36⁣′N 37°28⁣′E 12.6°N 37.467°E, respectively. The average annual rainfall in the area ranges from 880 to 1172 mm, while the average annual temperature is 19.7°C, with a range of 30.7°C–12.3°C. The region experiences two distinct seasons, the wet season from June to September and the dry season from October to May. The farming system in the area is of a mixed type [23].

### 2.2. Study Population

The study involved lactating dairy cows and calves from selected governmental and private dairy farms within the study area. These farms were selected based on the voluntary participation of their managers and accessibility. The age of the study animals was classified into the following categories: less than 1 year, 1–5 years, 5–10 years, and greater than 10 years using their dentition anatomy following the guidelines established by De-Launta and Habel [24]. The body condition of these animals was also evaluated according to the guideline principles [25]. The cattle herds on the studied dairy farm were noted for their small size, consisting of fewer than 25 animals. In contrast, a medium-scale farm is defined as having between 26 and 50 cattle, while a large farm is characterized by having more than 50 heads of cattle [2, 17].

### 2.3. Study Design

A cross-sectional study was conducted from March to August 2022 to determine the *Salmonella* isolates and their associated risk factors and antibiotic susceptibility patterns from lactating cows and calves in dairy farms in Gondar town, Ethiopia.

### 2.4. Sample Size Determination and Sampling Techniques

The sample size of this study was determined by using the study population formula given by Thrusfield [26]. *N* = (1.96)^2^*P*_exp_(1 − *P*_exp_)/*d*^2^, where *N* is the total number of sample size; *P*_exp_ is the expected prevalence; *d* is the absolute precision.

The sample size was determined based on previous studies [2] with a prevalence rate of 12.5%, a significance level of 5%, and a 95% confidence interval (CI), using the formula calculations above. This resulted in a required sample size of 170 study animals. To enhance accuracy, a 20% nonresponse rate was added to the calculated sample size. Consequently, a total of 200 study animals (161 dairy cows and 39 calves) and 32 bulk milk samples (one from each farm) were collected from 32 purposively selected dairy farms in the study area, chosen based on livestock availability and the willingness of farm owners. In total, 232 samples (200 fecal from the study animals and 32 bulk tank milk) were collected to isolate *Salmonella* and perform an antibiotic susceptibility test in the study area.

A simple random sampling using the lottery technique was used to select dairy cows and calves in dairy farms. These farms were classified into public and private facilities. After that, a quota was allocated to each farm in proportion to the number of dairy cows in each farm. Because the number of lactating cows was adjustable, we included all lactating cows of the study animals in this study.

### 2.5. Sample Collection and Laboratory Methods

#### 2.5.1. Questionnaire Survey

Information on the associated risk factors of the farm owners was gathered through a close-ended questionnaire survey for all 200 study animals. The questionnaire was initially developed in English and then translated into Amharic. The survey included questions regarding farm ownership, farm size, and cleanliness, as well as feed and water management. To ensure consistency, the translated questionnaire was independently back-translated into English by another person who was unaware of the original version.

#### 2.5.2. Sample Collection and Transportation

Fresh fecal samples were aseptically collected directly from the anus of healthy dairy cows and calves using disposable gloves and placed in sterile plastic bags. Similarly, milk samples were aseptically collected from the bulk containers of each farm using sterile universal bottle. Approximately 30 g of fecal samples from dairy cows and calves was placed in sterile containers, while about 25 mL of bulk milk was collected from the tank of each selected farm in sterile universal bottle. All samples were carefully labeled with information such as the collection date, source, and sample type. The samples were then transported in an icebox containing ice packs and analyzed at the Veterinary Microbiology Laboratory at the University of Gondar, Ethiopia.

#### 2.5.3. Isolation and Identification

*Salmonella* was isolated and identified following the protocols established by ISO-6579 [27] and the World Health Organization's Global Foodborne Infection Network [28]. Initially, the pre-enrichment was conducted by transferring a 3 g fecal sample and 25 mL of tank milk in separated sterile materials. From these, 27 mL of Buffered Peptone Water (BPW) was added to each sample. Their mixture was then agitated using a stomacher at a speed of 230 rpm for 1–2 min, followed by a 24-h incubation at 37°C. After incubation, the pre-enriched broth was thoroughly mixed, and 1 mL from each pre-enriched culture was transferred into a tube containing 10 mL of tetrathionate (TT) broth. Additionally, 0.1 mL of the broth was added to another tube with 10 mL of Rappaport (RV) broth, which was subsequently incubated for 24 h at either 37°C or 42°C. Finally, 10 *μ*L of the loop-full sample was inoculated onto xylose lysine deoxycholate (XLD) agar and brilliant green agar (BGA) derived from each selective enrichment broth (TT and RV) with the plates incubated for 24 h at 37°C.

The unique *Salmonella* colonies were maintained on nutrient agar, tryptic soy agar (TSA), and tryptone soy broth (TSB). The broth was stored in a deep freezer, while the TSA and TSB agar containing *Salmonella* were monitored daily to ensure their viability for biochemical testing. The pure cultures obtained from nutrient agar and TSA plates were subsequently inoculated into various biochemical confirmation media using an inoculating loop, which included Simmons citrate agar, methyl red (MR), Christensen's urea agar, lysine iron agar (LIA), Voges Proskauer (VP), and tryptone water [29].

#### 2.5.4. Antibiotic Susceptibility Tests

The antibiotic susceptibility of *Salmonella* isolates was assessed using the Kirby–Bauer disk diffusion method on Muller–Hinton agar (HiMedia CM0337, India) with the guidelines established by the National Committee for Clinical Laboratory Standards (NCCLS) [30, 31]. Pure isolated colonies on nutrient agar were picked with a wire loop, transferred to a tube containing 5 mL of saline water, and emulsified. The broth culture was then incubated at 37°C for 4 h until it reached the 0.5 McFarland turbidity standard. A sterile cotton swab was dipped into the suspension, and the bacteria were uniformly spread over the surface of a Muller–Hinton agar plate within a sterile safety cabinet. The plates were left at room temperature for 15 min to dry after which antibiotic discs containing known concentrations of antimicrobials were placed on the agar. The plates were incubated at 37°C for 24 h. Subsequently, a selection of 10 commonly utilized antibiotics was introduced which included ampicillin (10 *μ*g), amoxicillin–clavulanic acid (30 *μ*g), gentamycin (10 *μ*g), cefoxitin (5 *μ*g), nalidixic acid (30 *μ*g), ciprofloxacin (10 *μ*g), tetracycline (30 *μ*g), kanamycin (30 *μ*g), chloramphenicol (30 *μ*g), and sulfamethoxazole–trimethoprim (25 *μ*g). After a 24-h incubation period at 37°C, the diameters of the zones of inhibition were measured to the nearest millimeter. The recorded zones were classified as sensitive, intermediate, or resistant under CLS guidelines [30, 31].

### 2.6. Data Analysis

The Statistical Package for Social Sciences (SPSS) Version 25 was used to analyze the data. Additionally, descriptive statistics such as cross-tabulations, percentages, and frequencies were computed to describe the frequency of *Salmonella* within the potential associated risk factors. Binary logistic regression analysis was also performed to identify the possible risk factors related to the occurrence of *Salmonella* isolates. An odds ratio (OR) at a 95% CI was also used to calculate the strength of associations, and a *p* value ≤ 0.05 was regarded as statistically significant.

### 2.7. Ethics Approval and Consent to Participate

All procedures and animal care adhered to Federations of Animal Sciences Societies (FASS) [32] guidelines. This study was approved by the Animal Welfare and Ethical Review Committee of the Department of Veterinary Medicine at Woldia University in Ethiopia (Ref. No. WDU/CoA/VM/024/05/8/2024). All appropriate precautions were taken to reduce the pain endured by the animals engaged in this study. Notably, there were no known dangers or discomforts related to taking samples from the study animals. In addition, study participants provided oral consent.

## 3. Results

### 3.1. The Overall Prevalence of *Salmonella* Isolates

The overall prevalence of *Salmonella* isolates was 12.9% (30/232) across all samples. Of these, 11.5% (23/200) of the study animals and 21.9% (7/32) of the bulk tank milk samples tested positive for *Salmonella*. Among the study animals, 6.8% (11/161) of cows and 30.8% (12/39) of calves had fecal samples that tested positive for *Salmonella* in the study area (Table 1).

### 3.2. The Occurrence of *Salmonella* Isolates and Their Associated Risk Factors

The chi-square test analysis revealed a significant association (*p* value ≤ 0.05) between *Salmonella* isolates and factors such as the animal's age, farm size, bedding, and udder washing practices. However, the isolation rate of *Salmonella* showed no statistically significant difference (*p* value = 0.148) among samples collected from clean, medium-clean, and unclean farms. The cross-tabulation analysis also showed that the distribution of *Salmonella* across various farms indicated that 25.5% of cows and calves were fed with external feed sources, while the remaining 74.5% relied on on-farm resources. Regarding water sources, 62.2% of lactating cows and calves consumed clean water from piped sources, 33% used river water, and 4.5% depended on well water. Additionally, the assessment of dairy farm hygiene showed that 12% of the animals were housed in well-maintained clean farms, 42.5% in medium-clean farms, and the rest in poorly maintained farms. In the case of body conditions, medium (12.6%) and good (3.2%) body conditions were statically insignificant; the prevalence is higher in poor body conditions (13.8%) (Table 2).

The binary logistic regression analysis revealed a significant association between *Salmonella* and animal age (*p* value = 0.001). Animals under 1 year old (OR = 3.498) and those aged 1–5 years (OR = 1.165) were 3.45 and 0.16 times more likely to be exposed to *Salmonella*, respectively, compared to animals older than 10 years (OR = 3.498, CI = 0.587–20.824). On the other hand, animals aged between 5 and 10 years were 0.732 times less likely to be exposed (OR = 0.268) than those older than 10 years. Regarding farm size, larger farms exhibited a higher proportion of *Salmonella* exposure (25.9%) compared to small farms (9.9%). Consequently, large (OR = 1.035) and medium-sized farms (OR = 1.399) were 0.35 and 3.99 times more likely to have *Salmonella* exposure than small farms, respectively. In terms of bedding type, *Salmonella* was more prevalent in concrete bedding (18.8%) than in mud bedding (4.8%). Concrete bedding had 5.382 times higher odds of *Salmonella* occurrence compared to mud bedding (OR = 5.382, CI = 1.565–18.511). Furthermore, the likelihood of *Salmonella* exposure was significantly higher in cows both before (6.7 times) and after washing (4.8 times) compared to those whose udders were not washed (Table 3).

### 3.3. Antimicrobial Susceptibility Patterns of *Salmonella* Isolates

The antibiotic susceptibility profile of the *Salmonella* isolates showed 100%, 96.7%, and 26.6% resistance to ampicillin, tetracycline, and cefoxitin, respectively. Conversely, the isolates were 100%, 90%, and 66.7% sensitive to ciprofloxacin, kanamycin, or sulfamethoxazole–trimethoprim and amoxicillin–clavulanic acid, respectively. Additionally, 30% of the isolates demonstrated intermediate susceptibility to both nalidixic acid and chloramphenicol as shown in (Table 4).

## 4. Discussions

The overall prevalence of *Salmonella* isolates was 12.9% (30/232) from the total collected samples, which aligns with 9.5% in Jimma [33], 10.76% in Addis Ababa [8], 10.5% in Modjo town [17], 12.5% in Gondar town [2], and 12.9% in Hawassa town [34]. These similarities could be attributed to comparable dairy farming practices, environmental conditions, and the level of biosecurity measures implemented in these regions [35]. Conversely, this finding was higher than 4.4% in Adama and Modjo towns [6], 2.7% in Addis Ababa [7], 1.6% from Addis Ababa dairy products [36] from various regions of Ethiopia and 4% in Iran [18]. The higher prevalence of *Salmonella* observed in the current study may be attributed to factors such as differences in agroecological zones, inadequate husbandry practices and biosecurity measures, and temporal variations which predispose to *Salmonella* occurrences in the study area. On the other hand, this finding was also lower than the previous findings of 20% in Jimma [37], 21.3% in Bishoftu [4], 24.56% in Meki [38], 44.8% in Gondar [2], and 64.3% in Hawassa [34]. The lower prevalence rates observed in the current study compared to previous reports may be attributed to differences in laboratory techniques which this study utilized BGA for the isolation of *Salmonella* species, as recommended by the WHO [28] and ISO-6579 [27]. Additionally, variations in *Salmonella* prevalence rates across studies may result from factors such as farm contamination, differences in sample types, study populations, and the timing of sample collection [6, 39].

The fecal prevalence of *Salmonella* isolates in this study was 11.5%, aligning closely with findings from previous research, including 11.4% in Bahir Dar [40], 10.7% in Hawassa [34], 8.98% in Adama and Modjo [6], and 7.6% in Central Ethiopia [41]. However, this prevalence was substantially higher compared to earlier reports of 4.7% in Central Ethiopia [21], 3.7% in Asella [12], 2.97% in Bedele and Nekemte [42], and 2.7% in Addis Ababa [7]. Conversely, it was lower than reports of 20% in Jimma [37], 17.54% in Modjo [38], and 16% in Sebeta [43]. These discrepancies can be attributed to variations in animal management practices, environmental factors, and the widespread occurrence of *Salmonella* among the region's livestock population. The shedding of *Salmonella* in feces contributes to the contamination of water, soil, other animals, and feed, which are potential sources of infection [6, 12].

The prevalence of *Salmonella* isolates in bulk tank milk samples from the dairy farm was 21.8%, which is comparable to findings from previous studies, such as 19% in Modjo town [38] and 14.3% in Asella [12]. However, this finding was higher than earlier reports, including 0% in Meki town [38], 10% in Adama and Modjo towns [6], and 10% in Central Ethiopia [21]. These differences may be attributed to variations in sampling methods, microbiological testing protocols, farm biosecurity measures, milk hygiene practices, and the cleanliness of feed and water, all of which influence the level of *Salmonella* contamination in milk. Cattle are a known reservoir for *Salmonella* species, shedding the bacteria through feces, which can contaminate milk and dairy products, thereby creating a direct pathway for human infection [7]. Moreover, the occurrence of *Salmonella* isolates may also be linked to various host- and environment-related factors [6, 17, 34]. Host factors include age, breed, physiological state, feeding strategies, and vaccination status [44]. Environmental factors like hygiene and management practices, stocking density, type and quantity of feed, access to clean water, use of contaminated utensils, housing conditions, ventilation, animal movement, calving environment, and production facilities all contribute to the prevalence of *Salmonella* in different areas of Ethiopia [45]. Furthermore, these variations can also be attributed to differences in the agroecological location of cattle, farm management practices, stocking density, housing conditions, feeding habits, and the types of feed provided [12, 34].

A significant association was identified between the *Salmonella* isolates and the related factors of age, farm size, bedding, and the cleanliness of the animal's udder (*p* < 0.05). Furthermore, the presence of *Salmonella* isolates showed a significant association with cattle age, aligning with the findings of previous studies by Eguale [41] and Adeladlew et al. [40]. The variations in *Salmonella* isolates observed among different age groups can be attributed to the underdeveloped adaptive immune systems of young cattle and calves, along with their relatively low levels of protective microflora, which increase their susceptibility to the disease. Furthermore, the limited understanding of bovine immune function, combined with the lack of advanced tools for immunological studies, poses a significant challenge to achieving and maintaining optimal cattle health [46]. A significant association between the *Salmonella* isolates and the size of the farms is also consistent with earlier reports from Ethiopia [38, 40, 47]. Nevertheless, it contradicts the earlier findings [2, 7, 41] which indicated that the *Salmonella* isolates did not show statistical significance with the herd size of the dairy farm. These variations attributed to the increased herd size has led to overcrowded conditions, resulting in heightened animal-to-animal contact and stress among the animals, thereby facilitating the transmission of *Salmonella* among these cattle [41].

Housing conditions and udder washing were significantly related to this disease [40]. These findings might be attributed to poor environmental sanitation, insufficient udder preparation, and the use of unhygienic milk containers, all of which can contribute to this bacterial contamination of raw milk [48]. The previous reports in Gondar town [2] noted that the difference in water source for cattle showed a statistical association with the isolation rate for *Salmonella*. The risk of *Salmonella* shedding seems to vary by production system, housing type, general hygiene level, management type, and animal age [49]. The differences observed can be also attributed to factors such as hygiene and management practices, stocking density, availability of water supplies, the use of contaminated utensils, types of housing, ventilation, animal movement, and the production facilities in various regions which contribute to the prevalence of *Salmonella* in different areas of Ethiopia [12, 34].

The misuse of antibiotics in both human and veterinary medicine has led to the development of resistance in bacteria against commonly used therapeutic antibiotics [18]. The findings of this study indicate that all isolates exhibited complete resistance (100%) to ampicillin and 96.7% resistance to tetracycline. These results are consistent with previous study reports from Ethiopia [2, 8, 34, 40, 47]. On the other hand, the *Salmonella* isolates were 75% susceptible to tetracycline [40] and 42.6% resistant to tetracycline for the isolates in Gondar [48]. The level of this drug resistance observed in this study may be attributed to the inappropriate use of antibiotics in dairy farming practices, in contrast to the practices in the aforementioned developed countries. The differences observed in these studies may be linked to assertions of drug resistance, which arise from the availability of medications without a prescription. Additionally, poor sanitation in the surrounding areas plays a significant role in the emergence of resistant strains [16].

This current finding has shown sensitivity to ciprofloxacin (100%), kanamycin (90%), and sulfamethoxazole + trimethoprim (90%). The effectiveness of ciprofloxacin against *Salmonella* isolates from both calves and cows aligns with previous findings reported in Ethiopia [2, 7, 12, 17, 33, 38, 40, 47, 50]. Ciprofloxacin was also found to be 100% effective against all humans and cattle from *Salmonella* isolates in Sudan [51]. The sensitivity percentage of kanamycin (90%) is also related to the earlier findings of 100% [7] and 72.74% [33]. On the contrary, earlier studies indicated that 65% [47] and 82% [17] of the *Salmonella* isolates from dairy farms in Ethiopia exhibited resistance to kanamycin. The sensitivity of *Salmonella* isolates from dairy farms to sulfamethoxazole + trimethoprim (90%) aligns with previous reports indicating sensitivity rates ranging from 85.7% to 71.4% [2, 7, 33, 38, 47, 50]. These differences in antimicrobial resistance may constitute a worldwide threat to the efficiency of medicines for both preventing and treating illness and are caused by complex interacting variables at the interface of a multifactorial One Health System that connects clinical human, animal, and environmental aspects [40, 47, 52]. Furthermore, variations in the levels of antimicrobial resistance of *Salmonella* across various regions of the country may be linked to risk factors associated with the agent, including virulence, pathogenicity, infectiousness, and host specificity that are connected to the genetic makeup of *Salmonella* strains [33].

## 5. Conclusion and Recommendations

The high prevalence of *Salmonella* isolates in dairy cows and calves presents a potential public health threat, particularly for individuals who consume raw milk. Risk factors such as the animals' age, farm size, bedding materials, and udder washing practices were identified as statistically significant risk factors (*p* value ≤ 0.05) associated with *Salmonella* isolation. Regarding antibiotic susceptibility, all *Salmonella* isolates were resistant to at least one of the tested antibiotics, showing complete resistance to ampicillin while remaining sensitive to ciprofloxacin. Consequently, further research into the genetic characteristics of these isolates, especially the resistant strains, is essential to understand the mechanisms underlying antibiotic resistance and to effectively prevent related diseases.

## Figures and Tables

**Table 1:** Sample type distribution of *Salmonella* isolates in study dairy farms.

**Sample type**	**Total examined**	**Number of positive samples (%)**
Cow feces	161	11 (6.8%)
Calf feces	39	12 (30.8%)
Tank milk	32	7 (21.9%)
Total	232	30 (12.9%)

**Table 2:** Occurrence of *Salmonella* and its associated risk factors in the study area.

**Risk factor**	**Categories**	**No. of the tested animals**	**No. of positive with prevalence (%)**	**X** ^2^	**p** ** value**
Age	Less than 1year	39	12 (30.8%)	19.649	0.001
	1–5 years	45	5 (11.1%)		
	5–10 years	99	4 (4%)		
	Greater than 10 years	17	2 (11.8%)		

Body conditions	Poor	58	8 (13.8%)	2.520	0.284
	Medium	111	14 (12.6%)		
	Good	31	1 (3.2%)		

Ownership	Government	34	7 (20.6%)	3.324	0.068
	Private	166	16 (9.6%)		

Farm size	Small	111	11 (9.9%)	6.516	0.038
	Medium	62	5 (8.1%)		
	Large	27	7 (25.9%)		

Feed source	Feed from farm	149	17 (11.4%)	0.005	0.945
	Feed from other sources	51	6 (11.8%)		

Water source	Pipe water	125	15 (12%)	0.084	0.959
	River	66	7 (10.6%)		
	Well water	9	1 (11.1%)		

Farm hygiene	Poor	91	13 (14.3%)	3.818	0.148
	Medium	85	10 (11.8%)		
	Good	24	0 (0%)		

Farm cleanliness	Poor	75	10 (13.3%)	3.392	0.183
	Medium	102	13 (12.7%)		
	Good	23	0 (0%)		

Bedding	Concrete	96	18 (18.8%)	9.535	0.002
	Mud	104	5 (4.8%)		

Udder wash	Before	72	13 (18.1%)	6.977	0.031
	After	68	8 (11.8%)		
	No	60	2 (3.3%)		

	Total	200	23 (11.5%)		

**Table 3:** Binary logistic regression analysis of *Salmonella* and its significant risk factors.

**Risk factor**	**Categories**	**No. of animals examined**	**No. of positive with prevalence (%)**	**AOR**	**95% CI**
Age	Less than 1 year	39	12 (30.8%)	3.498	0.587–20.824
	1–5 years	45	5 (11.1%)	1.165	0.183–7.398
	5–10 years	99	4 (4%)	0.268	0.041–1.732
	Greater than 10⁣^∗^ years	17	2 (11.8%)	1	

Farm size	Small	111	11 (9.9%)	1	
	Medium	62	5 (8.1%)	1.399	0.361–5.429
	Large⁣^∗^	27	7 (25.9%)	1.035	0.207–5.176

Bedding	Concrete	96	18 (18.8%)	5.382	1.565–18.511
	Mud⁣^∗^	104	5 (4.8%)	1	

Udder wash	Before	72	13 (18.1%)	6.713	1.218–36.988
	After	68	8 (11.8%)	4.857	0.844–27.944
	No⁣^∗^	60	2 (3.3%)	1	

Total		200	23 (11.5%)		

⁣^∗^1 = reference.

**Table 4:** Antibiotic susceptibility test of *Salmonella* species isolates in the study area.

**Antimicrobials**	**Unit (*μ*g)**	**Status of antimicrobial against the isolates**		
		**S** ** (%)**	**I** ** (%)**	**R** ** (%)**
Ampicillin	10	0 (0.0)	0 (0.0)	30 (100)
Amoxicillin–clavulanic acid	30	20 (66.7)	8 (26.6)	2 (6.7)
Gentamycin	10	17 (56.7)	7 (23.3)	6 (20)
Cefoxitin	30	19 (63.3)	3 (10)	8 (26.7)
Nalidixic acid	30	15 (50)	9 (30)	6 (20)
Ciprofloxacin	5	30 (100)	0 (0.0)	0 (0.0)
Tetracycline	30	0 (0.0)	1 (3.3)	29 (96.7)
Kanamycin	30	27 (90)	1 (3.3)	2 (6.7)
Chloramphenicol	30	15 (50)	9 (30)	6 (20)
Sulfamethoxazole + trimethoprim	25	27 (90)	3 (10)	0 (0.0)

## Data Availability

The data can be obtained from the corresponding author upon request, as it is subject to ethical restrictions.
